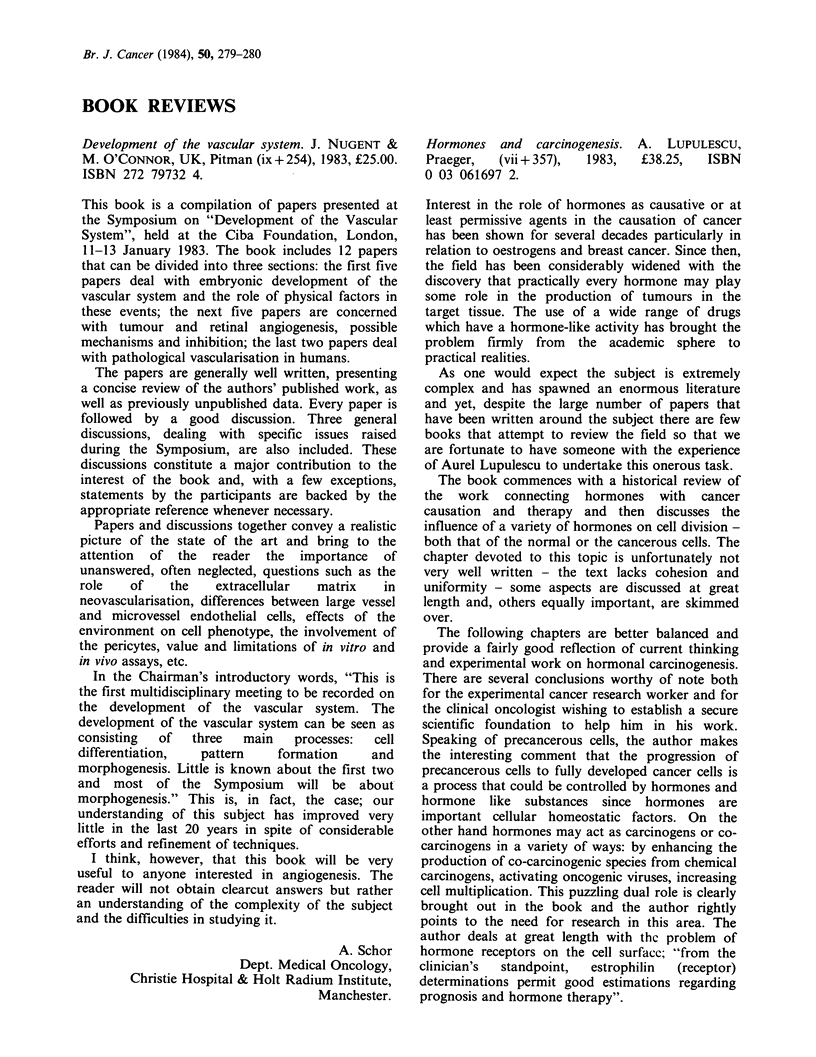# Development of the vascular system

**Published:** 1984-08

**Authors:** A. Schor


*Br. J. Cancer* (1984), **50**, 279–280

### BOOK REVIEWS

*Development of the vascular system.* J. NUGENT & M. O'CONNOR, UK, Pitman (ix+254), 1983, £25.00. ISBN 272 79732 4.

This book is a compilation of papers presented at the Symposium on "Development of the Vascular System", held at the Ciba Foundation, London, 11–13 January 1983. The book includes 12 papers that can be divided into three sections: the first five papers deal with embryonic development of the vascular system and the role of physical factors in these events; the next five papers are concerned with tumour and retinal angiogenesis, possible mechanisms and inhibition; the last two papers deal with pathological vascularisation in humans.

The papers are generally well written, presenting a concise review of the authors' published work, as well as previously unpublished data. Every paper is followed by a good discussion. Three general discussions, dealing with specific issues raised during the Symposium, are also included. These discussions constitute a major contribution to the interest of the book and, with a few exceptions, statements by the participants are backed by the appropriate reference whenever necessary.

Papers and discussions together convey a realistic picture of the state of the art and bring to the attention of the reader the importance of unanswered, often neglected, questions such as the role of the extracellular matrix in neovascularisation, differences between large vessel and microvessel endothelial cells, effects of the environment on cell phenotype, the involvement of the pericytes, value and limitations of *in vitro* and *in vivo* assays, etc.

In the Chairman's introductory words, "This is the first multidisciplinary meeting to be recorded on the development of the vascular system. The development of the vascular system can be seen as consisting of three main processes: cell differentiation, pattern formation and morphogenesis. Little is known about the first two and most of the Symposium will be about morphogenesis." This is, in fact, the case; our understanding of this subject has improved very little in the last 20 years in spite of considerable efforts and refinement of techniques.

I think, however, that this book will be very useful to anyone interested in angiogenesis. The reader will not obtain clearcut answers but rather an understanding of the complexity of the subject and the difficulties in studying it.

A. Schor
Dept. Medical Oncology,
Christie Hospital & Holt Radium Institute,
Manchester.